# The Crustacean Central Nervous System in Focus: Subacute Neurodegeneration Induces a Specific Innate Immune Response

**DOI:** 10.1371/journal.pone.0080896

**Published:** 2013-11-20

**Authors:** Paula Grazielle Chaves da Silva, Clynton Lourenço Corrêa, Sergio Luiz de Carvalho, Silvana Allodi

**Affiliations:** 1 Programa de Neurobiologia, Instituto de Biofísica Carlos Chagas Filho, Universidade Federal do Rio de Janeiro, Rio de Janeiro, Rio de Janeiro, Brazil; 2 Programa de Pós-Graduação em Morfologia, Instituto de Ciências Biomédicas, Universidade Federal do Rio de Janeiro, Rio de Janeiro, Rio de Janeiro, Brazil; 3 Faculdade de Medicina, Universidade Federal do Rio de Janeiro, Rio de Janeiro, Rio de Janeiro, Brazil; Universidade de São Paulo, Brazil

## Abstract

To date nothing is known about the subacute phase of neurodegeneration following injury in invertebrates. Among few clues available are the results published by our group reporting hemocytes and activated glial cells at chronic and acute phases of the lesion. In vertebrates, glial activation and recruitment of immunological cells are crucial events during neurodegeneration. Here, we aimed to study the subacute stage of neurodegeneration in the crab *Ucides cordatus*, investigating the cellular/molecular strategy employed 48 hours following ablation of the protocerebral tract (PCT). We also explored the expression of nitric oxide (NO) and histamine in the PCT during this phase of neurodegeneration. Three immune cellular features which seem to characterize the subacute phase of neurodegeneration were revealed by: 1) the recruitment of granulocytes and secondarily of hyalinocytes to the lesion site (inducible NO synthase- and histamine-positive cells); 2) the attraction of a larger number of cells than observed in the acute phase; 3) the presence of activated glial cells as shown by the round shaped nuclei and increased expression of glial fibrillary acidic protein. We suggest that molecules released from granulocytes in the acute phase attract the hyalinocytes thus moving the degeneration process to the subacute phase. The importance of our study resides in the characterization of cellular and biochemical strategies peculiar to the subacute stage of the neurodegeneration in invertebrates. Such events are worth studying in crustaceans because in invertebrates this issue may be addressed with less interference from complex strategies resulting from the acquired immune system.

## Introduction

Wallerian degeneration is an extensively studied phenomenon which occurs a few days after nervous injury in vertebrates [1]. Although nervous degeneration has not been much studied in the last years, it is known from papers published some decades ago, that it is slower in invertebrates and lower vertebrates than in higher vertebrates [2], [3]. Indeed, long-term survival of axons for many days and even for months has been shown in lower vertebrates and invertebrates [2], [3], [4], [5], [6]. This makes sense if we consider the entire animal kingdom: the rapid degeneration is probably the exception, not the rule [2]. Two hypotheses related to glial cells, based mainly on studies conducted in crustaceans, may explain the long survival of axons in invertebrates after degeneration. One presumes that glial cells surrounding the axons produce the necessary proteins to maintain their function and these proteins are transferred to the enucleated axons [7], [8]. The other presupposes that glial cells “donate” their nuclei to the severed axons [9], [10], [11].

The activation of glia [12], [13] and the recruitment of immunological cells are events related to nervous degeneration [14], [15], [16], [17], [18]. In addition, blood cells are important components that integrate the nervous and immune systems during inflammatory processes: after injuries to the nervous tissue [19], [20], blood cells are attracted to the injury site and produce different substances, such as histamine and NO, which are common to both innate and acquired immune systems (the immune system present in vertebrates) [21], [22], [23], [24], [25].

It is known that in vertebrates the first cells of the immune system that are attracted to the site of a nervous injury by trauma during the acute phase are neutrophils [26], [27], [28] and, in the subacute and chronic phases, lymphocytes among other cells [27], [29], [30]. However, the cellular/molecular strategy employed in invertebrates in response to nervous injury is still unknown. The few morphological reports on crustacean degenerating nerve fibers did not reveal the cellular immunological response for the acute, subacute, and chronic phases [2], [3], [4], [5], [6]. In fact, the most relevant available clues are the results published by our group reporting the presence of hemocytes at the chronic [31] and acute phases of the lesion [32].

Therefore, to further understand the events that occur in the subacute stage of nervous degeneration in invertebrates, we attempted to investigate the cellular/molecular strategy employed by the crab *Ucides cordatus* at 48 hours following ablation of the protocerebral tract (PCT). We also explored the expression of inducible NO synthase (iNOS) and histamine in the PCT during the subacute phase of nervous degeneration. The study of such events in invertebrates is important because this issue may be addressed with less interference from complex strategies resulting from the acquired immune system. The PCT was chosen because it is composed of axons and glial cells [33], [34], and therefore suitable for degeneration studies [31].

## Materials and Methods

### Animals and Ethics Statement

Adult male crabs (*Ucides cordatus*) with carapace lengths between 6.5 and 8.0 cm were used in this study. These animals were collected from Duque de Caxias, State of Rio de Janeiro, Brazil, and maintained in the laboratory under standardized light conditions (12 h/12 h light/dark cycle), temperature 24°C for no longer than 10 days before they were killed.

All procedures adopted in this study, including the location where the animals were captured, were performed after approval by the National Environmental Committee (Certificate # 14689-1/IBAMA/2008, permission to use the animals # 2440408), and by the Ethics Commission on Research Animals of the Centro de Ciências da Saúde, Universidade Federal do Rio de Janeiro (protocol DHEICB 005).

The crabs were fed once a day with commercial pellet food (Alcon Garden Basic Sticks). Each animal was anesthetized by cooling for 30 min, before we produced a lesion in the PCT by eyestalk ablation and collected the hemolymph as detailed below. Only animals with carapace having no obvious signs of damage were used in our study. The PCT was chosen given its simplicity and easy accessibility for neurodegeneration study purposes.

### Eyestalk ablation

Five crabs had their eyestalks ablated unilaterally, as described in Figure 1 of [31], in order to cause degeneration of the distal stump of the PCT. After 48 h, the remaining part of the PCT was dissected in 4% paraformaldehyde (PA) and 2.5% glutaraldehyde in 0.1 M phosphate-buffered crustacean saline (crust-PBS), pH 7.4, prepared according to [35]. The PCTs of the contralateral eyestalks were used as controls.

### Hemolymph collection

Ten crabs had their hemolymph collected under normal conditions (5 animals) and 48 h after ablation (5 animals). The hemolymph was collected in an anticoagulant solution (1∶3 v/v: 0.1 M glucose, 15 mM sodium citrate tribasic, 13 mM citric acid, 10 mM EDTA, 0.45 M sodium chloride, pH 7.4) by inserting a 5-mL syringe into the articulation of the first pair of thoracic appendages of each crab. The hemocytes were plaqued on glass coverslips for 30 min (for cell adhesion), and fixed with 4% PA or carbodiimide +4% PA (for histamine labeling) prepared in ideal osmolality for another 30 min, followed by a rinse with crust-PBS. This procedure was followed by immunohistochemistry and histochemistry.

### Histochemistry and routine light microscopy

Plaqued hemocytes and PCT frozen sections were processed for histochemistry in order to identify macrophages/microglia-like cells (to be labeled with isolectin B4) among the circulating hemocytes and the injured PCT. Fixed cells and tissues were rinsed with crust-PBS. The slides with PCT or hemocytes were incubated with *Bandeiraea simplicifolia* isolectin B4 (IB4, diluted to 5 µg/mL; Sigma) conjugated to fluorescein isothiocyanate prepared in 1 mM crust-PBS-calcium, at 4°C, overnight. On the following day, the material was washed with crust-PBS and mounted with anti-fading mounting medium. The specificity of the staining was checked by saturation of the lectin binding sites with D-(1)-galactose (300 mg/mL). The negative control of the reaction consisted of omitting the lectin. Some slides were used for hematoxylin and eosin staining.

### Immunohistochemistry

Normal and injured PCTs of five crabs were fixed in 4% PA for 4 h, cryoprotected in sucrose overnight, embedded in OCT (Optimal Cutting Temperature; Tissue Tek®), and sectioned in a cryostat (Leica CM 1850). Five-micrometer sections were collected on gelatinized slides, washed in crust-PBS, 3 times for 5 min each time. For the immunohistochemical reactions, cryosections obtained as described above were washed in distilled water, permeabilized with 0.3% Triton X-100/crust-PBS for 15 min, washed again with crust-PBS, and incubated overnight with the primary antibody prepared in a blocking solution composed of 3% bovine serum albumin in crust-PBS. The monoclonal antibody used was against glial fibrillary acidic protein (GFAP – anti-rabbit IgG; Sigma), diluted at 1∶100, which labels glial cells, including in invertebrates (Florim da Silva et al., 2004); iNOS (anti-rabbit; Sigma), diluted at 1∶100, histamine (anti-rabbit; Sigma) diluted 1∶100, and phospho-histone 3 diluted at 1∶50 (anti-rabbit; Sigma). The secondary antibody was Alexa 546 (goat anti-rabbit) diluted at 1∶600. DAPI (4′,6-diamidino-2-phenylindole) was added as a nuclear staining. The slides were mounted in crust-PBS/n-propylgallate and observed under a Zeiss Axioskop 2 fluorescence microscope equipped with a color camera (Media Cybernetics, model Evolution™ MP). The controls consisted of omitting the incubation in the primary antibody.

### Transmission electron microscopy

Unilateral segments of the tracts from five crabs were removed and used as controls. After 48 h the remaining tracts were dissected and fixed in 4% PA and 2.5% glutaraldehyde in crust-PBS, pH 7.4, prepared according to [35]. Then they were washed in 0.1 M phosphate buffer (with adjusted salinity). The samples were postfixed in 1% osmium tetroxide plus 0.8% potassium ferrocyanide and 5 mM calcium chloride in 0.1 M cacodylate buffer (pH 7.4) for 2 h (in the dark). After this procedure, the samples were rinsed in 0.1 M phosphate buffer, and then block-stained in 1% uranyl acetate overnight. The dehydration was performed in a graded series of acetone up to 100%. The specimens were then embedded in Polybed 812 (Polyscience) resin. After polymerization for 48 h at 60°C, the resulting blocks were sectioned using a RMC MT 6000 ultramicrotome. Semithin sections (400–500 nm) were stained with 1% toluidine blue, and observed by light microscopy to evaluate the orientation and the quality of the sections. Ultrathin sections (60–70 nm) were collected, stained with 2% uranyl acetate for 20 min and 1% lead citrate for 3 min. The sections were analyzed by the transmission electron microscope Jeol JEM-1011 belonging to the Rudolf Barth Electron Microscopy Platform of the Oswaldo Cruz Institute/Fiocruz, and by the transmission electron microscope LM 906-Carl Zeiss belonging to the Laboratório de Microscopia Eletrônica Prof. Luiz Henrique Monteiro Leal – LABMEL/Instituto de Biologia Roberto Alcântara Gomes/Universidade do Estado do Rio de Janeiro.

### Statistical analysis

The statistical analyses were performed to quantify cell nuclei in the control and injured PCTs. We used paired analysis by Student's test (between two groups: normal and injured animals; n = 5 crabs/group). We also used paired analysis by Student's test to compare numbers of cell nuclei in two different regions of the same PCT 48 h after lesion (n = 5 crabs). The confidence interval was 95%, with an accepted alpha value of 5% (p<0.05). The analyses were carried out using the Prism Graph Pad 4.0 statistical software.

## Results

### Injured PCT shows more cells than control

**Figure 1:**
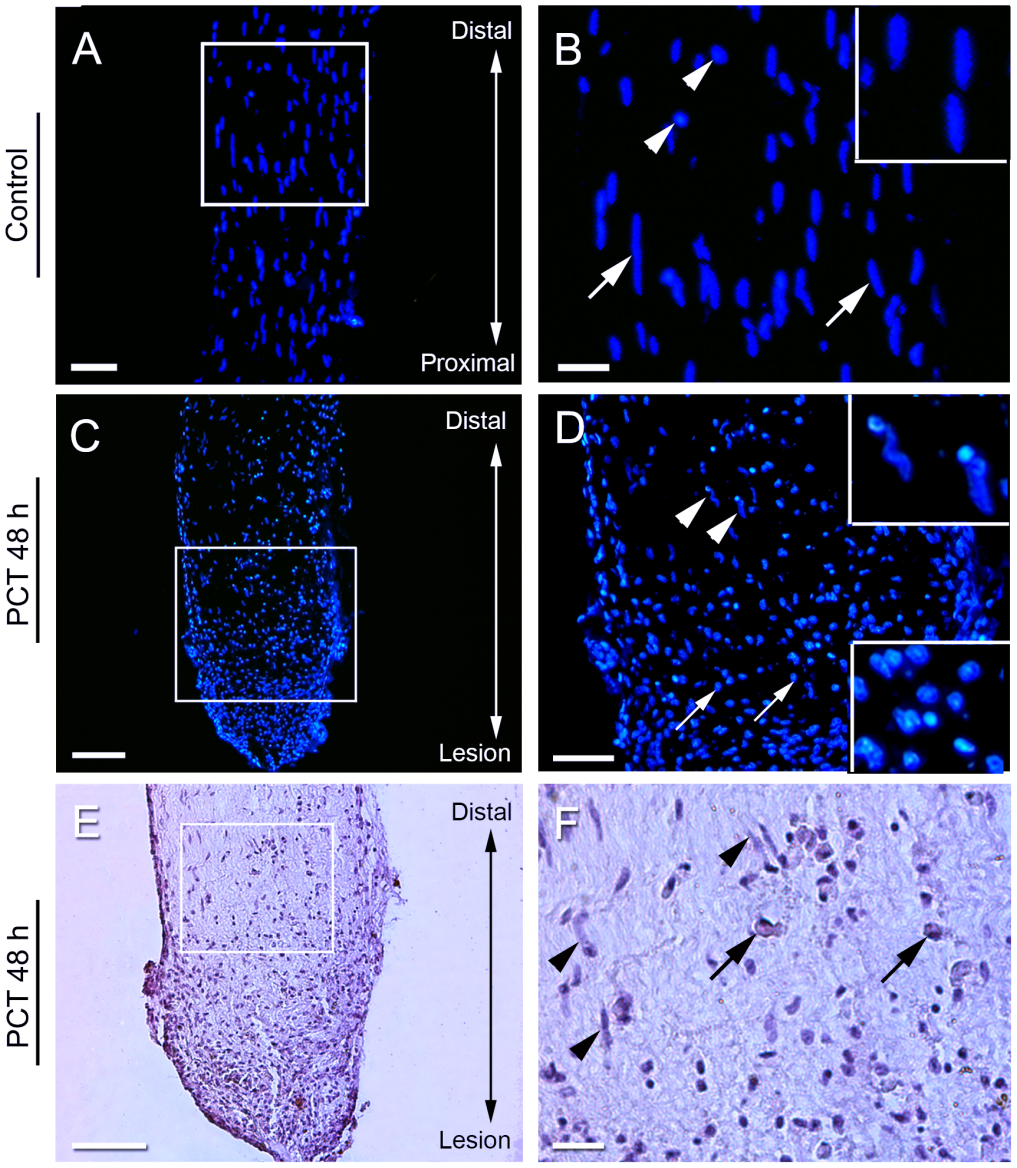
General morphology of the PCT. (A–D) Nuclear staining with DAPI. (A, B) Control. (B) Higher magnification of the region within the square in A. Note the cells showing elongated nuclei (arrows) and symmetric distance between each other (insert), resembling glial cells. The arrowheads denote an unusual cell type with round nuclei. (C, D) 48 h after lesion: the damaged area has a high density of cells (bottom of the image). (D) The majority of the cells have round nuclei (D – bottom insert); note also cells with elongated nuclei in the region distal to the lesion (arrows – top insert). (E, F) Hematoxylin and eosin staining showing the morphology of hemocytes 48 h after lesion. F is a higher magnification of the square sampled in E. Note in F the hemocytes (arrows) and resident glial cells (arrowheads) within the PCT. Scale bars: A, C, E = 100 µm; B, D, F = 50 µm.

To reveal the morphology and the distribution of the cell nuclei along the PCT of *Ucides cordatus* before (control PCT) and after lesion (injured PCT), histological sections were stained with hematoxylin and eosin and DAPI. Cells spread along the whole control PCTs with many elongated and flattened nuclei, resembling glial cells, and positioned at regular distance from one another were observed (Figure 1A and B). In contrast, injured PCTs showed a large number of cells close to the lesion site (Figure 1C and D). Nuclei of these cells were round, small, and often grouped or aligned (Figure 1E and F). Because no labeling for phospho-histone 3 was detected in these cells (data not shown) it is plausible that they are not proliferating *in situ*. In the region distal to the lesion, cells showed irregular morphology as compared with the controls. Forty-eight hours after the lesion the connective tissue surrounding the PCT was very evident (Figure 1E).

**Figure 2:**
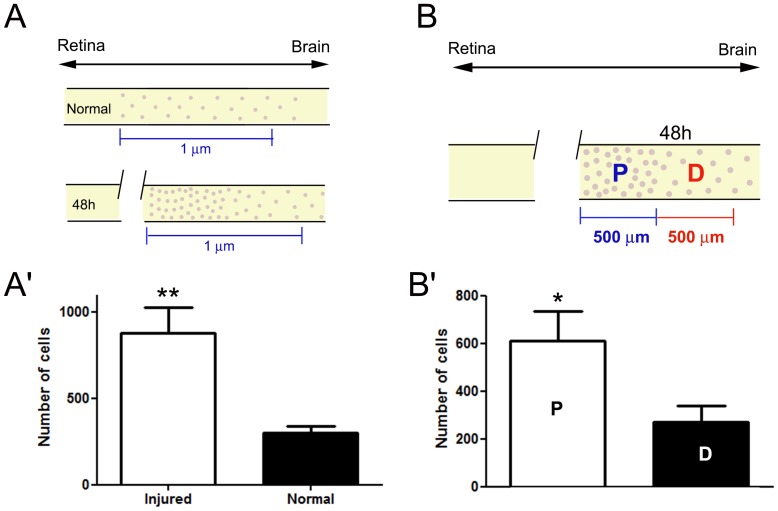
Density of cells in the PCT increases after injury. (A) Schematic illustration of both normal and injured PCT showing the cellular arrangement along the tract, including the quantified area (1 µm). The anatomical position of the tract is represented by a double-sensed arrow on the top (retina – brain). (A′) 48 h after injury, the distal part of the tract had approximately three times as many cells as the control group. Student t test revealed a significant difference between the two groups (n = 5 crabs/group; p<0.005). (B) Schematic illustration of the injured tract divided into two regions (500 µm each): proximal (P) and distal (D) to the lesion. Most cells are grouped in the damaged area 500 µm from the injury. In contrast, region D maintains the same cellular density as seen in the control. (B′) Statistical analysis shows twice as many cells in region P as in D. Student t test revealed a significant difference between the two groups (n = 5 crabs/group; p<0.05). Asterisks denote significant differences between groups.

Statistical analyses confirmed a greater number of cells in the injured PCT when compared with the control group (Figure 2A and A′). When we compared the region proximal to the injury (region P) with the region distant to the lesion (region D), we observed that the region P had almost twice as many cells as region D (Figure 2B and B′).

### Hyaline and granular hemocytes are attracted to the lesioned area

**Figure 3:**
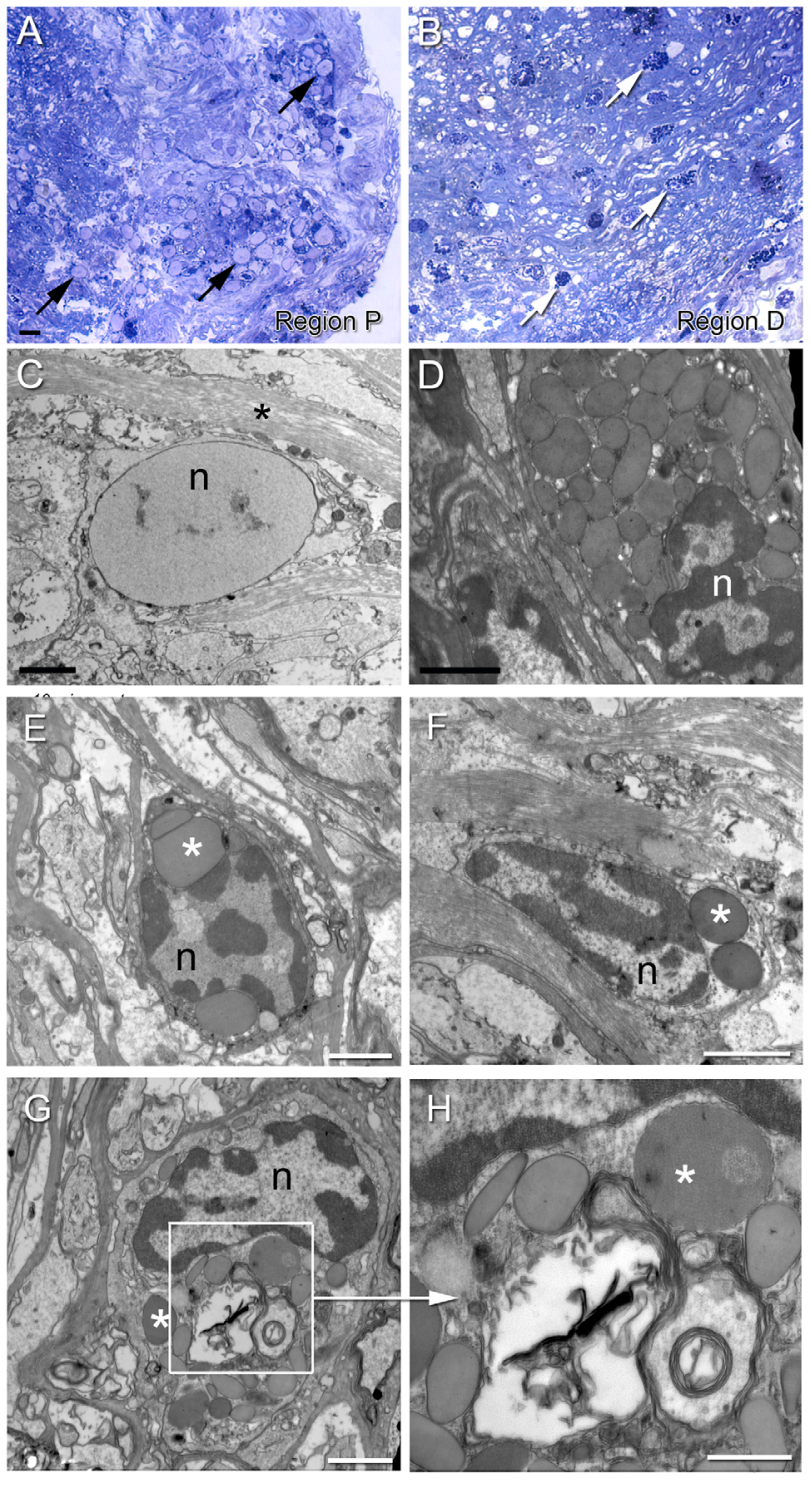
PCT attracts two different types of cells to the lesion site. (A, B) Semithin sections of injured PCT stained with toluidine blue. (A) Region P (proximal) is surrounded by cells with typical morphology: round shape, high nucleus/cytoplasm ratio, and no granules within the cytoplasm (arrows), suggesting hyaline cells. (B) Granular hemocytes (arrows) infiltrating into the nerve fibers of the tract after eyestalk ablation – region D (distal to the lesion). Only granular cells are observed in this region intermingled with nerve fibers. (C, D) Ultrathin sections of regions P and D, respectively. (C) Hemocyte, resembling a hyaline cell, with round shape and no granules in the scarce cytoplasm. The eccentric nucleus (n) has abundant euchromatin displaying the same features as the cells shown in (A). The asterisk indicates a nerve fiber. (D) Typical granular hemocyte with numerous electron-dense granules occupying the whole cytoplasm. The nucleus (n) shows an irregularly shaped membrane and heterochromatin in the periphery. (E, F) Semigranular hemocytes infiltrated into the injured tract – region D (distal to the lesion). A small number of electron-dense granules (asterisks) occupy the whole cytoplasm; the eccentric nuclei (n) have irregular/flattened shape with abundant heterochromatin in the periphery, which is not seen in hyaline hemocytes. (G, H) Granular hemocytes are easily seen in region D and most of them show double-membrane vesicles (square) within the cytoplasm suggesting phagocytosis. This is better seen in D (higher magnification). Electron-dense granules (asterisks) with different shapes and sizes surround these vesicles. Scale bars: A, B = 10 µm; C–G = 2 µm; H = 1 µm.

According to our previous study granular hemocytes are attracted to the lesion site 24 h after PCT ablation [32]. Here we investigated whether the same cells were recruited to the lesion site in a subacute stage of degeneration. Semithin (Figure 3A and B) and ultrathin (Figure 3C and D) sections showed two different types of hemocytes in the PCT 48 h after lesion. Hyaline cells, with no granules and a higher nucleus/cytoplasm ratio, as classified by [32], were frequently found in region P, mainly in the outer limit of the PCT. The majority of the cells displayed a high amount of heterochromatin (Figure 3C).

Region D showed granular/semigranular hemocytes within the tract (Figure 3B). These hemocytes were clearly distinguishable from resident glial cells by the typical morphology and organization of the glial cells in the PCT. Ultrathin sections revealed that granulocytes have electron-dense granules with different sizes, occupying almost the entire cytoplasm, and irregular nuclei with peripheral euchromatin (Figure 3D). Different morphologies of the semigranular hemocytes were also seen in region D after injury. They were surrounded by the nervous fibers and had fewer granules in the cytoplasm than the granulocytes (Figure 3E and F). In addition, some hemocytes revealed many vesicles containing membranes and electron-dense material resembling cellular debris (Figure 3G and H), typical of phagocytosis.

### Hemocytes attracted to the lesion site express histamine

**Figure 4:**
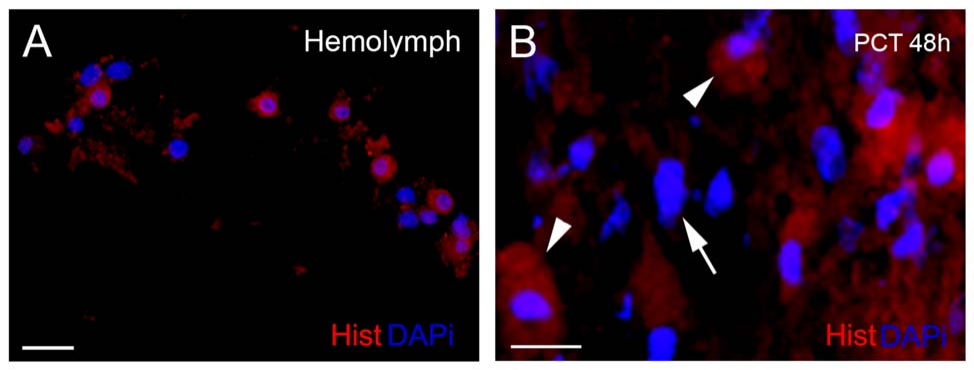
Circulating hemocytes and PCT cells are labeled for histamine. (A) Histamine-positive cells are red in the hemolymph; and the nuclei, DAPI stained, are in blue. (B) Injured PCT (48 h after eyestalk ablation) showing cells labeled for histamine (red) and DAPI (blue). The majority of histamine-positive cells contain granules (arrowheads) and round nuclei. No labeling was seen in the elongated nuclei (arrow) of the cells with morphology of glial cells. Scale bars: A = 20 µm; B = 10 µm.

During neurodegenerative processes, the participation of mast cells has been described due to production of pro-inflammatory mediators, such as histamine [36], [37]. In this paper, we observed that histamine can be detected in the circulating hemocytes and in cells recruited to the PCT 48 h after injury (Figure 4). No cell with elongated nucleus (i.e., glial cell) showed histamine labeling.

### iNOS expression is increased after injury

**Figure 5:**
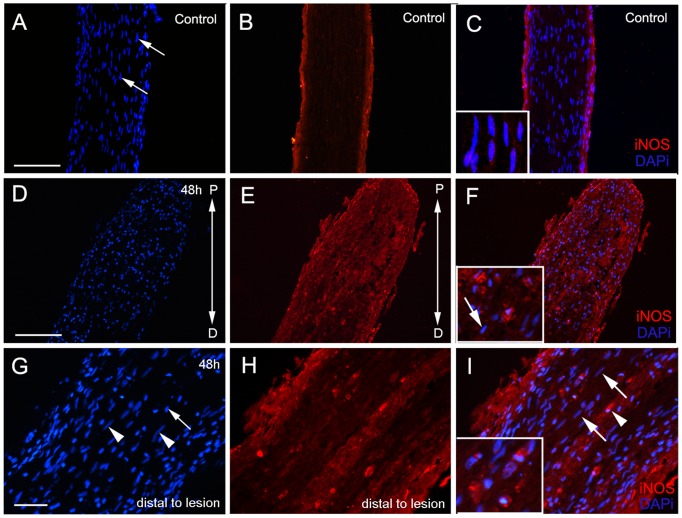
Expression of iNOS in the PCT 48 h after lesion. (A, B, C) Control animals. (A, D, G) DAPI stained cells; (B, E, H) iNOS immunoreacted (red); (C, F, I) Merge. No cells from control PCTs were labeled for iNOS (C – insert). (D–F) Region P (proximal) of the tract observed after DAPI nuclear staining (D), after iNOS reaction (E), and the merged image (F). The insert shows a higher magnification of the double-labeled cells. (G–I) Region D (distal) of the tract observed after DAPI staining (G), after iNOS labeling (H), and the merged image (I) revealing numerous hemocytes containing granules (arrowhead), infiltrating into the tissue. No labeling was seen in the elongated nuclei (arrows) of the cells with morphology of glial cells. The insert for I shows a higher magnification of the labeled hemocytes. Scale bars: A–I = 100 µm.

To study different aspects of the inflammatory reaction, we verified glial activation, microglia/microphage response, and iNOS expression during the degeneration of the PCT. Immunohistochemistry for iNOS revealed that more cells were labeled close to the injury site than in the control (Figure 5). Between cells labeled for iNOS it was possible to identify a few resident cells (with features of glial cells) which were seen distally to the lesion (Figure 5C′ and C′′). Apparently, no resident cells were labeled with iNOS.

**Figure 6:**
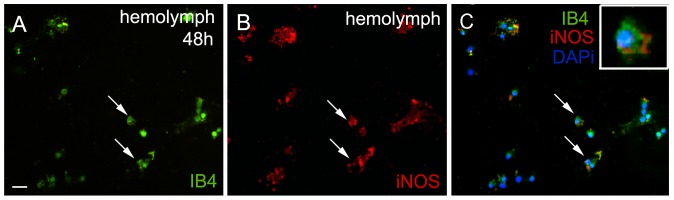
iNOS and IB4 expression are colocalized in circulating hemocytes. Hemolymph was collected from five crabs, 48(A –green) and iNOS (B – red). Only hemocytes with granules, semigranular/granular cells, showed double-labeling (insert). Scale bar: A–C = 10 µm.

In vertebrates, macrophages and microglia are potentially able to produce NO [38]. Considering that NO is increased in the lesion site and that macrophages/microglia produce it, we wanted to know whether the granular cells, which appear in high quantities in the lesion, were analogous to vertebrate macrophages/microglia (revealed by the labeling with IB4). Our data show some circulating hemocytes with granules co-labeled for IB4 and iNOS (Figure 6). iNOS production was not observed in PCT cells from crabs that were not submitted to ablation.

**Figure 7:**
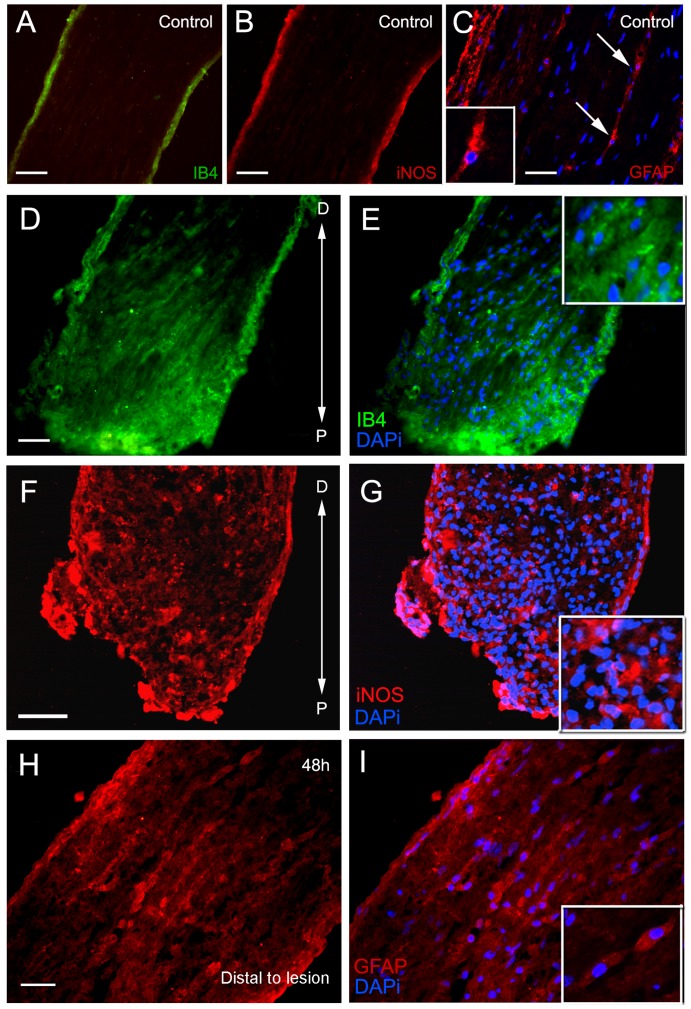
Lesioned area of the PCT has a high expression of iNOS, IB4, and GFAP. (A, B, C) Controls. (A) IB4; (B) iNOS; (C) GFAP. (C) GFAP-positive cells (arrows) with round nuclei and elongated cytoplasm are seen between nerve fibers of the control PCT. (D, E) Region P (bottom of the image) showing IB4 cells (insert in E) most intensively labeled. Distal to the lesion (top of the image), IB4 labeling is weak and few cells are seen. (F, G) iNOS expression in the lesion site. Note that iNOS and IB4 expression are mainly located in the PCT edge. (H, I) Region D of the tract showing several positive glial cells labeled for GFAP. They have a different morphology from resident glial cells and are seen aligned along the tract (insert). Scale bars: A, B = 50 µm; C = 40 µm; D, E = 50 µm; F, G = 40 µm; H, I = 30 µm.

Because the inflammatory response during nervous system injuries triggers immune responses [14], [17], [18] and glial activation [12], [13], we wanted to investigate how glial cells were disposed in the PCT together with recruited hemocytes, and the nature of their morphology in the subacute phase (or 48 h after the lesion). We observed more GFAP-, IB4-, and iNOS-positive cells concentrated in the lesion site when compared with the controls. Additionally, GFAP-positive cells showed different nuclear morphology from resident glial cells. They showed round nuclei and were aligned along the tract (Figure 7).

## Discussion

The subacute phase of nervous degeneration following injury in invertebrates has not been a much explored subject, particularly whether such a stage would involve immune cellular events. In this study we revealed three immune cellular features which seem to characterize the subacute phase of nervous degeneration in the invertebrate studied: 1) The recruitment of mainly granular or semigranular hemocytes and, secondarily, hyalinocytes to the lesion site. Using iNOS and histamine labeling we were able to confirm that the recruited hemocytes were active. 2) The recruitment of a larger number of cells than observed in the acute phase, also to lesion site [32]. 3) The presence of activated glial cells revealed by the round shape of their nuclei and increased expression of GFAP.

In vertebrates, after acute central nervous system injuries there is an inflammatory response involving microglial activation and infiltration of granular cells such as macrophages, and neutrophils [27], [29], [36]. It is known that these cells may produce both NO and histamine [39], [40]. Despite lacking acquired immunity, invertebrates use the same molecules (iNOS and histamine) produced by defense mechanisms triggered by vertebrate macrophages, mast cells, and neutrophils: hemocytes from other invertebrates have been shown to produce histamine (ascidians in [41]) and NO (ascidians in [42], horseshoe crab in [43]). Because expression of histamine and iNOS was seen in both the circulating hemocytes and in the PCT 48 h after eyestalk ablation, we suggest that the circulating hemocytes are the cells recruited to the PCT. Moreover, we identified by specific markers that the recruited hemocytes producing iNOS are macrophage/microglia-like cells and those producing histamine are another subtype of granule cells present in the lesion site.

Because iNOS and histamine were more expressed in the lesion than in the controls we may speculate that they participate in the tissue strategy used for attracting circulating hemocytes and activation of glial cells as observed herein. Since only granulocytes (granular/semigranular cells), and not hyalinocytes, are present in the acute phase [32] we suggest that histamine and NO released from granulocytes in the acute phase attract the hyalinocytes, thus moving the degeneration process to the subacute phase.

**Figure 8:**
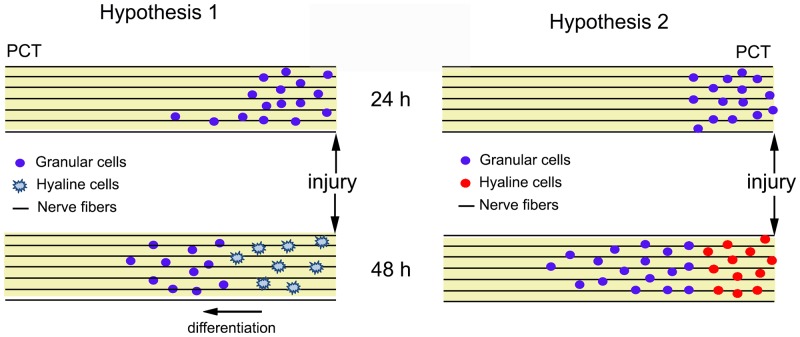
Schematic diagram of the hypotheses for the source of hemocytes to the injured PCT. Hypothesis 1: Granular/semigranular (dark blue) are the first cell type to be attracted to the injured tract. In sequence, hyaline cells (light blue) are also attracted and can differentiate in granular hemocytes as they infiltrate into the nerve fibers (due to several stimuli, such as histamine and iNOS). Hypothesis 2: Granular/semigranular hemocytes (blue) are attracted to the lesion site. Following the time course of the lesion, they infiltrate into the tract. Then, in a subacute stage, hyaline cells (red) are also attracted to the injury. In both hypotheses, we can identify two different types of cells in the PCT.

Hyalinocytes are considered less differentiated than granulocytes [44] and as such they could replenish the lesion site, differentiate, and perform defensive roles, such as phagocytosis [45], [46], [47] of cellular debris as observed here. This should be the case if hemocytes are all differentiated from a same progenitor cell type [48], [49].We may suggest this case to be one hypothesis for the source of hemocytes to the injured PCT (Figure 8). In this context semigranular cells may constitute an intermediate stage of maturation, since less mature hemocytes are acquiring granules, or they may constitute a more mature stage undergoing degranulation in response to a challenge [50].

However, because other authors have suggested that hemocytes are originated by diverse precursor cell types [51], [52] - and this may be another hypothesis for the source of hemocytes to the injured PCT - they would arrive at different times, characterizing the acute and subacute stages: the first to appear being the granulocyte and in a second moment, the hyalinocyte (Figure 8). Although we observed a large number of cells close to the lesion site we could not see labeling with anti-phospho-histone 3 (data not shown), therefore the cells did not divide *in situ*, they most probably migrated to the lesion site. Further studies will be necessary to establish which of the proposed hypotheses actually help explain the cellular strategy used by invertebrates for the neurodegeneration response observed. An interesting speculation is that because iNOS and histamine are highly conserved molecules [40], [41], [42], [43] it is possible that they are involved in nervous degeneration responses of similar kinds in other organisms. Once again, more research will be needed to establish this suggestion.

## Conclusion

Inflammation and autoimmunity are two principal vertebrate immunological responses that can compromise the function of multiple organs and tissues, including the CNS [53]. Considering that invertebrates, and crustaceans in particular, only possess the innate response [49] – and therefore there is not a specific cell with immune memory – perhaps this is the mechanism they developed allowing neurodegeneration to occur in such a delayed period when compared with vertebrates [4], [5], [31]. To conclude, the importance of our study resides in the characterization of the cellular and biochemical strategies peculiar to the subacute stage of the neurodegeneration response in invertebrates.
